# Gender and the Sex Hormone Estradiol Affect Multiple Sclerosis Risk Gene Expression in Epstein-Barr Virus-Infected B Cells

**DOI:** 10.3389/fimmu.2021.732694

**Published:** 2021-09-08

**Authors:** Jeremy T. Keane, Ali Afrasiabi, Stephen D. Schibeci, Nicole Fewings, Grant P. Parnell, Sanjay Swaminathan, David R. Booth

**Affiliations:** ^1^Centre for Immunology and Allergy Research, Westmead Institute for Medical Research, University of Sydney, Sydney, NSW, Australia; ^2^BioMedical Machine Learning Lab (BML), The Graduate School of Biomedical Engineering, UNSW SYDNEY, Sydney, NSW, Australia; ^3^Department of Medicine, Western Sydney University, Sydney, NSW, Australia

**Keywords:** Epstein-Barr virus (EBV), multiple sclerosis, sex-bias, eQTL, estrogen, estradiol

## Abstract

Multiple Sclerosis (MS) is a complex immune-mediated disease of the central nervous system. Treatment is based on immunomodulation, including specifically targeting B cells. B cells are the main host for the Epstein-Barr Virus (EBV), which has been described as necessary for MS development. Over 200 genetic loci have been identified as increasing susceptibility to MS. Many MS risk genes have altered expression in EBV infected B cells, dependent on the risk genotype, and are themselves regulated by the EBV transcription factor EBNA2. Females are 2-3 times more likely to develop MS than males. We investigated if MS risk loci might mediate the gender imbalance in MS. From a large public dataset, we identified gender-specific associations with EBV traits, and MS risk SNP/gene pairs with gender differences in their associations with gene expression. Some of these genes also showed gender differences in correlation of gene expression level with Estrogen Receptor 2. To test if estrogens may drive these gender specific differences, we cultured EBV infected B cells (lymphoblastoid cell lines, LCLs), in medium depleted of serum to remove the effects of sex hormones as well as the estrogenic effect of phenol red, and then supplemented with estrogen (100 nM estradiol). Estradiol treatment altered MS risk gene expression, LCL proliferation rate, EBV DNA copy number and EBNA2 expression in a sex-dependent manner. Together, these data indicate that there are estrogen-mediated gender-specific differences in MS risk gene expression and EBV functions. This may in turn contribute to gender differences in host response to EBV and to MS susceptibility.

## Introduction

Multiple Sclerosis (MS) is an autoimmune disorder in which the myelin sheath of neurons in the central nervous system is damaged by immune cells (1). It is a complex disorder involving a strong genetic component and a number of highly implicated environmental factors. To date, over 200 MS susceptibility risk loci have been identified which are estimated to account for up to 48% of the genetic contribution to MS susceptibility (2). The genes associated with these loci largely function in immune cells, including T and B cells, monocytes, NK cells and microglia (2). Epstein-Barr virus (EBV), lack of sun exposure, vitamin D deficiency, and smoking are all environmental factors with different degrees of evidence (3). Notably, all of these environmental risk factors have effects on the immune system (3).

The gammaherpesvirus EBV, one of the most substantial environmental risk factors for MS, infects more than 90% of the global population and has been identified as necessary but insufficient on its own to cause MS (4). Nearly all patients with MS are seropositive for EBV (5, 6). The risk for MS increases in people with high anti-EBV antibody titers (7) or a history of the EBV syndrome infectious mononucleosis (IM) (8), and fluctuations in antibody titer are associated with relapses (9). Moreover, in a study of 1047 clinically isolated syndrome (CIS) cases, an episodic condition that precedes MS, only one patient was seronegative for EBV antigens (10). The virus has been postulated to affect MS pathogenesis across the MS disease course, from pediatric-onset MS to the relapsing-remitting and progressive forms of MS, and as such, the therapeutic targeting of EBV in MS patients has received attention (11). The use of EBV-specific cytotoxic CD8+ T cell therapy has provided promising results in an MS patient, reducing disease activity and intrathecal immunoglobulin production (12). This indicates both the potential for such treatments in MS and the role of EBV in disease activity.

At the genomic level, EBV is implicated with MS susceptibility *via* binding of the EBV transactivating protein EBNA2 at multiple MS risk loci (13), further implicating EBV infection in MS pathogenesis. We previously reported that 47 risk loci are associated with gene expression in EBV infected B cells, referred to as lymphoblastoid cell lines (LCLs) (14, 15). We also reported the over-representation of MS risk genes in host genes to be highly correlated with EBV DNA copy number in LCLs, while non-EBV associated diseases did not have such an association (15), suggesting an association between MS risk genes and the EBV life cycle. In addition, we found that the expression of ZC3HAV1, an MS risk gene with antiviral function, is dependent on the MS risk SNP in EBV infected B cells but not in uninfected B cells, highlighting the link between MS and EBV survival in the host (16). Despite this, surprisingly little is known about the molecular processes by which EBV may drive MS development.

The predominance of MS among females has been increasing in recent decades. Reports in the 1930s suggested a male predominance (17), but in the 1980s a female predominance of approximately 2:1 was largely reported (18–20), which has further increased to almost 3:1 (21–23). It is believed that the increase of MS among women has occurred too quickly to be attributable to changes in genetic composition and is likely to be due to rapid changes in lifestyle and environmental factors (3). These include differences in smoking (24–26), and obesity rates among women (27, 28). Perhaps more significantly, rapid changes in modern diets and reproductive behavior have occurred, factors that alter the levels of sex hormones.

The increased risk of MS in females is not seen before puberty, during pregnancy, or after menopause (20). Females are generally protected from MS during pregnancy, particularly during the third trimester, when estrogen levels are at their highest, and are at increased risk postpartum when estrogen levels have dropped (20). This tolerance and breach of tolerance is likely mediated by changes in sex hormones, particularly estrogens (29). This hormone exerts effects on all immune cells through estrogen receptor-dependent and -independent mechanisms. The experimental autoimmune encephalomyelitis (EAE) mouse model for MS supports this notion, finding that estradiol protects against disease *via* anti-inflammatory mechanisms (30–32), including through B cell mechanisms (33). However, despite all this, clinical trials of estrogens in MS have been unsuccessful (34).

Several other EBV-associated diseases also have disproportionately affected females. Two disorders with strong EBV association, systemic lupus erythematosus (SLE) and rheumatoid arthritis (RA), have female:male ratios of approximately 9:1 and 4:1, respectively (35). Other conditions with less definitive evidence for an EBV association include primary biliary cirrhosis, autoimmune thyroid disease, and Sjögren’s syndrome, with female:male ratios of approximately 10:1, 8:1, and 9-20:1, respectively (13, 36–40).

As EBV-associated disorders disproportionately affect females, and the genetic risk factors are effectively equal among the sexes, it is possible that a sex-dependent pathogenic response to EBV may contribute to susceptibility or progression of these diseases. The seroprevalence of EBV is the same among both sexes (41), however, as with most viruses, EBV antibody titers are generally higher in females (42, 43).

It is plausible that the host response to EBV may be involved in the development of MS, as mentioned above, and that this differing response between males and females is reflected in the different risk for MS. This could be mediated by sex hormone augmentation of the host EBV response and EBV infected B cell functions. A gender effect has been reported in the correlation of age of IM and the development of MS (44). We reasoned that a sex-dependent host response to EBV and the expression of MS risk genes in EBV infected B cells could be affecting MS pathogenesis and may contribute to the gender bias in the disease.

We analyzed the eQTL (expression quantitative trait loci) effects of MS risk loci in a European cohort of LCLs, identifying several risk loci with sexual dimorphism in the expression patterns of their associated genes. We also showed that Estrogen Receptor 2 (ESR2) correlates with EBV traits differentially in males and females. Hence, we further reasoned that this gender bias could be mediated by sex hormones. We tested this hypothesis first by using LCLs cultured in serum-free medium to remove estrogen and other agents with estrogenic effects, and in serum-free medium supplemented by estrogen. We anticipated that the estrogenic effects of serum would be lost in serum-free medium but would be restored by estradiol treatment. Our findings support our hypothesis that MS risk loci/genes and EBV traits are affected by donor gender and responsive to estrogens.

## Materials and Methods

### Calculation of Gender Specific eQTL Effects for MS Risk Loci in LCLs

Genotype data on 196 of the 201 GWAS identified non-HLA MS risk SNPs (2) in a European population were available from the 1000 Genome project (45) and the 216 genes proximal to these MS risk SNPs were extracted from the latest MS GWAS (2). The expression level of these genes in the LCLs derived from the same European individuals with genotype data was acquired from the GEUVADIS dataset (46). The MatrixEQTL R package (47) was used to estimate the eQTL effect for 229 MS risk SNP:gene pairs in 187 female and 171 male LCLs both separately and combined (358 LCLs). All 229 MS risk SNP:gene pairs tested for eQTL associations are described in **Supplementary Table 1**.

### Correlation of MS Risk Genes With Sex Hormone Receptors, EBV Genes and DNA Copy Number

The processed and normalized RNA-seq based host gene expressions (MS risk and sex hormone receptor genes) for 464 LCLs including 216 male LCLs and 248 female LCLs were obtained from GEUVADIS dataset (46). The processed and normalized RNA-seq based EBNA2 gene expression were obtained from EBV portal database (48). The estimated EBV DNA copy number for matched 433 LCLs samples (201 male and 232 female LCLs) were also obtained (49). To test the correlation between these elements, a Spearman’s rank-order correlation test was performed using R software on data for donor matched LCLs.

### Generation and Culture of LCLs

Blood was collected from healthy individuals with informed consent (Westmead Hospital Human Research Ethics Committee Approval 1425). Ficoll-Paque Plus (VWR International) was used to isolate peripheral blood mononuclear cells (PBMCs) as previously described (41). The generation of LCLs was carried out as previously described (14). Briefly, fresh or frozen PBMCs were incubated for 1 hr at 37°C with supernatant from the EBV B95-8 cell line, after which the cells were suspended in RPMI-1640 medium (Lonza) containing 10% fetal bovine serum (FBS, Sigma) and 2 mM L-glutamine (Life Technologies). The cells were plated at densities of 5 × 10^6^ cells per well in 48-well plates. The medium was supplemented weekly until the cells were expanded into a 25 cm2 flask. Expanded LCLs were cryopreserved in 10% DMSO (MP Biomedical) 50% FBS and RPMI-1640.

### Weaning of LCLs From Estrogenic Medium to Serum-Free Medium and Estradiol Treatment

All LCLs were cultured in typical growth medium for LCLs which consisted of RPMI-1640 (Lonza) supplemented with 10% FBS and 2 mM L-glutamine, which in this study is termed SCM (serum-containing medium). No antibiotics were added to any cultures. To remove the effect of FBS and the estrogenic effects of the pH indicator phenol red (50, 51) contained in RPMI-1640, the cells were weaned from SCM to a serum-free medium (SFM) which is phenol red-negative. The SFM consisted of X-VIVO 15 phenol red-free medium, (Lonza) and other additions (**Supplementary Table 2**). The weaning protocol used for all experiments, with the exception of the proliferation assay (outlined in *LCL Proliferation Rate*) is a modified version of that recommended by the manufacturer. Cell viability was checked for all LCLs prior to and during the weaning process by Trypan blue staining. Cells with a viability of 85% or above were considered acceptable. Briefly, the cells were weaned by demi-depletion of the medium with SFM over 9 days (on days 0,2,4,6). On day 7 the medium was either demi-depleted with SFM or with SFM containing 200 nM estradiol (to provide a final concentration of 100nM estradiol). 48 hr after the final demi-depletion (+/- estradiol), the cells were collected and prepared for analysis. Estradiol (as 17β-estradiol, Sigma Australia) was dissolved in ethanol just prior to use as previously described (52). We used estradiol (E2, or 17β-estradiol) in this study as it is the major form of endogenous estrogen in humans (53). A concentration of 100 nM estradiol was chosen based on prior studies (including those in LCLs) which corresponds to pregnancy level (54–57).

### RNA Extraction and cDNA Synthesis

Total RNA was isolated from LCLs using a Bioline Isolate II RNA Mini Kit (Bioline, U.K.) according to the manufacturer’s instructions. After treatments or incubation periods, cells were washed in DPBS and resuspended in 100 μL RLY Buffer provided in the kit. The samples were snap-frozen and stored at -80°C until required. Samples were thawed on ice and 100 μL of RLY Buffer and 2 μl of TCEP was added to samples and vortexed vigorously. The remaining steps for RNA extraction followed the manufacturer’s instructions. RNA was checked for quality and quantified using a NanoDrop 2000 Spectrophotometer (Thermofisher). cDNA was synthesized using qScript cDNA SuperMix (Quanta Biosciences).

### Gene Expression Profiling

For the detection of MS risk genes, 3 µL of diluted cDNA was used to detect gene expression of MS risk genes in duplicate by real time PCR using predesigned TaqMan gene expression assays (Life Technologies, Carlsbad, CA, USA) (**Supplementary Table 3**) and TaqMan Universal Master Mix II, with UNG, according to the manufacturer’s instructions. Gene expression was calculated using the 2^-ΔΔCT^ method as previously described (58), using RPL30 as the reference gene. Wilcoxon matched-pairs signed rank test (two-tailed) was performed using GraphPad Prism 8.

### Viral Gene Expression

For the detection of expression of EBV encoded genes EBNA2 and LMP1, 3 µL of diluted cDNA used with SYBR primer sets and Takara SYBR Pre-Mix Master Mix. Forward and Reverse primers (**Supplementary Table 4**) were used at a final concentration of 0.2 μM with 6 µL of SyBr mix. Wilcoxon matched-pairs signed rank test (two-tailed) performed using GraphPad Prism 8.

### EBV DNA Copy Number Measurement

For detection of EBV DNA copy number, cells were removed from culture after the treatment periods, washed with DPBS, and the pellets stored at -80°C. DNA was extracted from cells using a QIAamp DNA Blood Mini Kit (Cat.No. 51106) according to the manufacturer’s instructions. EBV DNA copy number was detected by Quantitative PCR using a previously described primer set and probe for an EBV genome-specific repeat region to detect EBV copy number (49). Real-time PCR was performed on a Biorad CFX384 qPCR System (Biorad) (49). A primer set for a single copy gene (**Supplementary Table 3**) was used as a reference to account for any DNA concentration variation between samples. The relative EBV DNA copy number was calculated as relative to the single copy gene the using the 2^-ΔΔCT^ method as previously described (58). Wilcoxon matched-pairs signed rank test (two-tailed) was performed using GraphPad Prism 8.

### LCL Proliferation Rate

A modified weaning protocol was used for the preparation of a proliferation assay as the reagent used, Cell Trace Violet (CTV, Life Technologies), does not effectively work over a prolonged period such as the 9 days used for other assays in this study. For this assay, the LCLs were prepared as before prior to day 0. On day 0, the LCLs were first labelled with CTV at a final concentration of 5 mM and incubated at 37°C for 10 mins. Quenched by adding bovine serum albumin (BSA) to a concentration of 0.5% w/v, the cells were then washed twice with PBS. 2 × 10^6^ LCLs were cultured with SCM or SFM. Unlike the other weaning process, in this case demi-depletion took place daily on days 1,2, and 3. The estradiol treated group were demi-depleted with SFM 200 nM estradiol on days 2 and 3. On Day 4 cells were harvested, washed with chilled PBS containing 0.05% sodium azide, pelleted at 300 x g (5 min), then fixed with 0.5 ml 1% paraformaldehyde and ran on FACSCanto II flow cytometer (BD). Median fluorescence intensity (MFI) of CTV was analyzed using FlowJo, with each sample compared to their Day 0 background values. Wilcoxon matched-pairs signed rank test (two-tailed) was performed using GraphPad Prism 8.

### MS Risk SNP Genotyping

For genotyping in the Westmead cohort, DNA was extracted from whole blood samples using Qiagen QIAamp DNA Blood Mini Kit (Qiagen). Samples were genotyped for MS-associated SNPs using Taqman Assays (**Supplementary Table 5**) and Taqman Genotyping Master Mix (Thermofisher/Life Technologies) according to the manufacturer’s instructions.

## Results

### Many MS Risk SNP eQTLs in LCLs Are Affected by Gender

**Figure 1:**
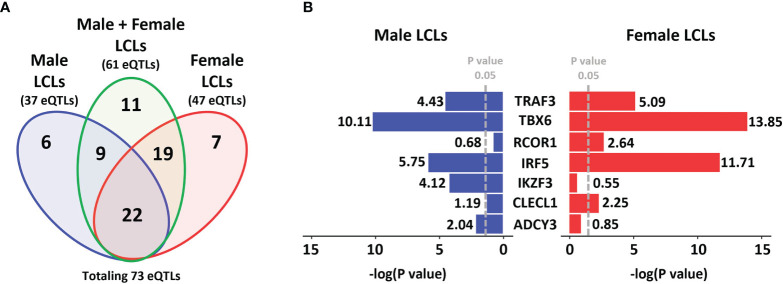
MS risk SNP eQTLs profile in LCLs demonstrates gender dimorphism. A total of 196 non-HLA MS risk SNPs were investigated to determine if they were eQTLs in LCLs (using a cut-off of p < 0.05) in the GEUVADIS dataset and those eQTLs identified were further investigated for gender dimorphism. **(A)** Data was divided into three sets, Male LCLs (blue), Male + Female LCLs (the entire cohort, green), and Female LCLs (red). A total of 37 eQTLs were detected in the Male set, 61 eQTLs were detected in the Male + Female set, and 47 eQTLs were identified in the Female set, with a total of 73 unique eQTLs occurring across the three sets. A total of 11 eQTLs were identified only in the Male + Female set. **(B)** Of the other 62 eQTLs, the 7 SNP:gene pairs with the strongest gender bias in eQTL effects in LCLs are presented. The GEUVADIS LCL cohort consisted of 358 European samples using RNA-seq including 187 female and 171 male LCLs.

To determine if any MS risk SNPs are sex-biased eQTLs, we interrogated the eQTL effect of MS risk SNPs (using a cut-off of p < 0.05) from the GEUVADIS dataset, based on three sets, which consisted of LCLs of both genders together (combined cohort), male LCLs and female LCLs (**Figure 1A**). From a total of 196 non-HLA MS risk SNPs, 73 were identified as eQTLs in LCLs in at least one of the three sets: 61 in the combined cohort, 37 in male LCLs, and 47 in females. Eleven eQTLs were identified in the combined cohort that were not eQTLs in either the male or female sets. From the remaining 62 SNPs, the seven SNP/gene pairs with the largest sex differences in the eQTL effect size were selected (**Figure 1B**). Two further SNP/gene pairs were included in the study due to sex differences in eQTLs detected in other study cohorts. Sex biased eQTLs were detected for ZC3HAV1 and CD40 in the MRCE (n = 950) and MRCA (n = 206) LCL cohorts (59–62). Our preliminary work on our LCL cohort (n = 42) also confirmed a sex biased eQTL for the MS risk SNP associated with ZC3HAV1, with opposite directions of this effect between male and female LCLs (male LCL p < 0.0266, female LCLs p < 0.005, **Supplementary Figure 1**). A total of 9 MS risk genes TBX6, ADCY3, TRAF3, CLECL1, RCOR1, IKZF3, IRF5, CD40 and ZC3HAV1 associated with 8 risk SNPs were selected for further investigation. All 73 unique MS risk SNPs with eQTL effects are described in **Supplementary Table 6**.

### Expression of Estrogen Receptor 2 Is Correlated With EBV Latency III Traits

**Figure 2:**
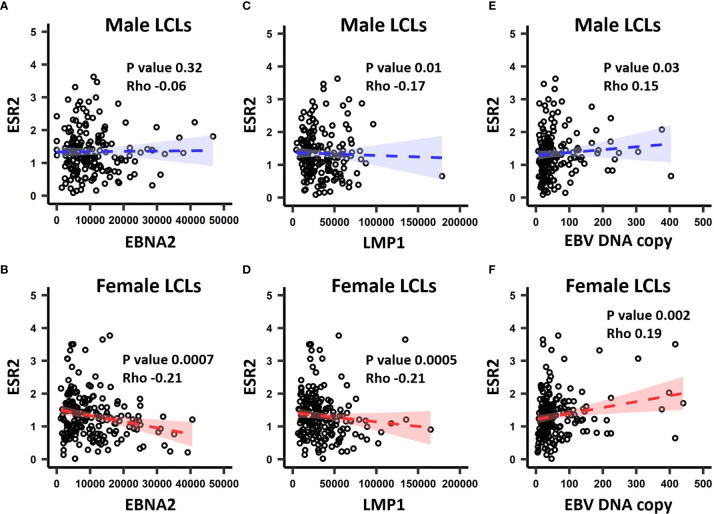
The correlation between ESR2 expression level and EBV latency III traits. ESR2 correlation with EBNA2 in **(A)** males and **(B)** females. Correlation between ESR2 expression level and LMP1 expression level in males **(C)** and in **(D)** females. Correlation between ESR2 expression level and EBV DNA copy number in males **(E)** and in **(F)** females. The RNA-seq based expression levels for ESR2, EBNA2 and LMP1 were obtained from the GEUVADIS study and EBV Portal for 216 male LCLs and 248 female LCLs. The estimated EBV DNA copy number and ESR2 expression level for 201 male and 232 female LCLs were used to estimate the correlation between ESR2 and estimated EBV DNA copy number. The correlations were calculated using Spearman’s rank correlation coefficient for donor matched LCL samples.

In the GEUVADIS cohort the LCLs from males and females were cultured in the same medium, yet demonstrated a gender effect of risk alleles on gene expression. This suggests that the LCLs have gender specific differences (for example, due to epigenetic effects) reflecting their gender of origin, and that this difference is maintained in culture. As the growth medium contains sex hormones and estrogen analogs, we hypothesized that these molecules could drive these differences. To determine if sex hormones might affect LCL phenotypes, we tested if expression of sex hormone receptors was correlated with the EBV latency III genes EBNA2 and LMP1 (n = 464 for both), in addition to EBV DNA copy number (n = 433) in the GEUVADIS cohort (see *Materials and Methods*). We tested for correlation of these markers against the expression of Estrogen Receptors 1 and 2 (ESR1, ESR2), the Progesterone Receptor (NR3C3), and the Androgen Receptor (AR). Among these, only ESR2 significantly correlated with the latency III traits (**Figure 2**, **Supplementary Table 7**), while NR3C3 expression was not detected in LCLs. EBNA2 expression was negatively correlated with ESR2 expression in female LCLs (rho -0.21, p < 0.0007) but not in male LCLs (rho -0.06, p < 0.32) (**Figure 2**). LMP1 expression was negatively correlated with ESR2 in both female and male LCLs, but much more so in females (rho -0.21, p < 0.0005 for females and rho -0.17, p < 0.01 for males, **Figure 2**). EBV DNA copy number was positively correlated with ESR2 expression, especially in females (rho 0.19, p < 0.002 for females and rho 0.15, p < 0.03 for males, **Figure 2**).

### Expression of MS Risk Genes Correlates With Estrogen Receptor 2 and EBV Latency III Traits

**Figure 3:**
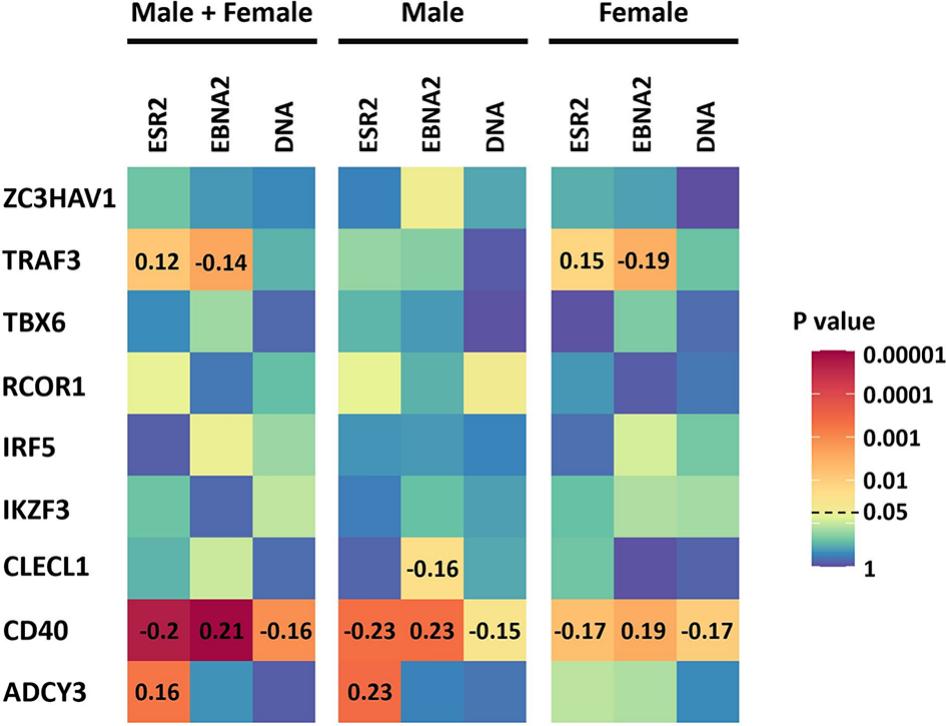
The correlation of the expression of the 9 MS risk genes in this study with Estrogen receptor 2 (ESR2) and the EBV latency III traits EBNA2 and EBV DNA copy number (DNA) of LCLs of known donor gender. ESR2, EBNA2 and EBV DNA copy number correlation with the expression of the 9 MS risk genes in male and female LCLs combined (Left), in male LCLs (center), and in female LCLs (right). The correlations were calculated using Spearman’s rank correlation coefficient. Heatmap shows the significance of the correlations (p value). For those correlations of statistical significance (p value less than 0.05), the Spearman’s correlation coefficient value (rho) is represented with numbers in the corresponding cells. The RNA-seq based expression levels for ESR2, EBNA2 and LMP1 were obtained from the GEUVADIS study and EBV Portal for 216 male LCLs and 248 female LCLs. The estimated EBV DNA copy number and ESR2 expression level for 201 male and 232 female LCLs were used to estimate the correlation between ESR2 and EBV DNA copy number.

As ESR2 expression was strongly correlated with latency III traits, dependent on gender, and we had previously identified sexual dimorphism in associations of MS risk SNPs with gene expression, we next tested for correlations of expression of these MS risk genes with ESR2 and latency III traits. Of the nine genes, four were correlated with either ESR2, EBNA2, or EBV DNA copy number (**Figure 3**, **Supplementary Table 8**). The most highly correlated gene was CD40. CD40 negatively correlated with ESR2 (rho -0.20, p < 0.000008) in male and female LCLs combined, as well as female (rho -0.17, p < 0.005) and male LCLs separately (rho -0.23, p < 0.0004). CD40 also negatively correlated with EBV DNA copy number in male and female LCLs combined (rho -0.16, p < 0.0007), female (rho -0.17, p < 0.009) and male LCLs (rho -0.15, p < 0.02). CD40 positively correlated with EBNA2 in male and female LCLs combined (rho 0.21, p < 0.000004), female (rho 0.19, p < 0.002) and male LCLs (rho 0.23, p < 0.0004). Furthermore, the correlation of CD40 with these elements was stronger in male LCLs than female. TRAF3, CLECL1 and ADCY3 demonstrated a gender effect, with TRAF3 correlating positively with ESR2 in female LCLs but not male (rho 0.15, p < 0.01 for females and rho 0.09, p < 0.16 for males), and negatively correlating with EBNA2 in female but not male LCLs (rho -0.19, p < 0.02 for females and rho -0.08, p < 0.19 for males). CLECL1 was slightly negatively correlated with EBNA2 in male LCLs but not female (rho -0.16, p < 0.02 for males and rho -0.003, p < 0.95 for females). ADCY3 positively correlated with EBNA2 in male LCLs but not female (rho 0.23, p < 0.0004 for males and rho 0.1, p < 0.11 for females, **Figure 3**).

### Estradiol Affects EBV Traits

**Figure 4:**
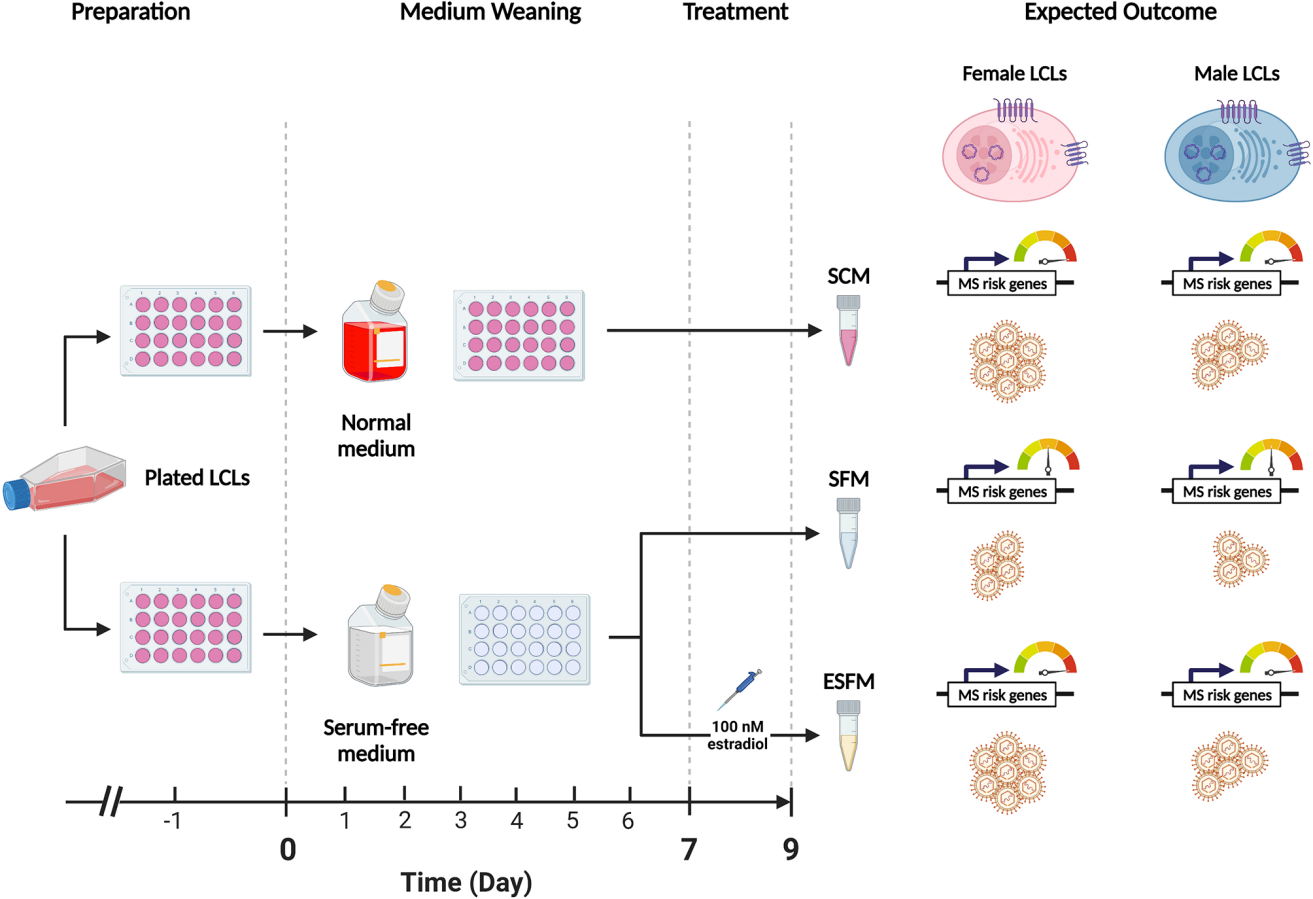
Overview of the weaning and estradiol treatment protocol used in this study. The standard medium for lymphoblastoid cell lines (LCLs) contains phenol-red, a pH indicating dye which has estrogenic effects. Fetal bovine serum (FBS) is also added to a final concentration of 10%-15% (v/v) to encourage the growth of cells but contains various hormones and cytokines which are confounding for the purposes of this study. This protocol weaned healthy LCLs from normal medium (RPMI-1690 containing phenol red and FBS 10%) to serum-free medium (SFM), which does not contain phenol red, over a 9-day period to remove the estrogenic effects of the medium. Preparation: proliferating LCLs cultured in normal medium were selected for study. One day prior to the start of the weaning protocol (Day -1), healthy LCLs were plated in 24-well plates and supplemented with normal medium. Medium Weaning: on Days 0,2,4 and 6 the medium was demi-depleted either with normal medium (top) or with serum-free medium containing no estrogenic agents. Treatment: on Day 7 the LCLs were demi-depleted as before, with estradiol added to one SFM group at a final concentration of 100 nM (estradiol-containing serum-free medium, ESFM). 48 hours later (Day 9) the cells were harvested for collection for three sets, SCM, SFM, and ESFM. On day 9 the cells were harvested for analysis. Expected Outcome: we hypothesized that the removal of estrogenic effects from the medium, (SCM to SFM) would affect the expression of the MS risk genes of interest and with EBV traits (symbolized by LCLs) in a gender-dependent manner. We further hypothesized that the reintroduction of estradiol would restore these effects. The dial indicates the expression of MS risk genes sensitive to estrogen.

**Figure 5:**
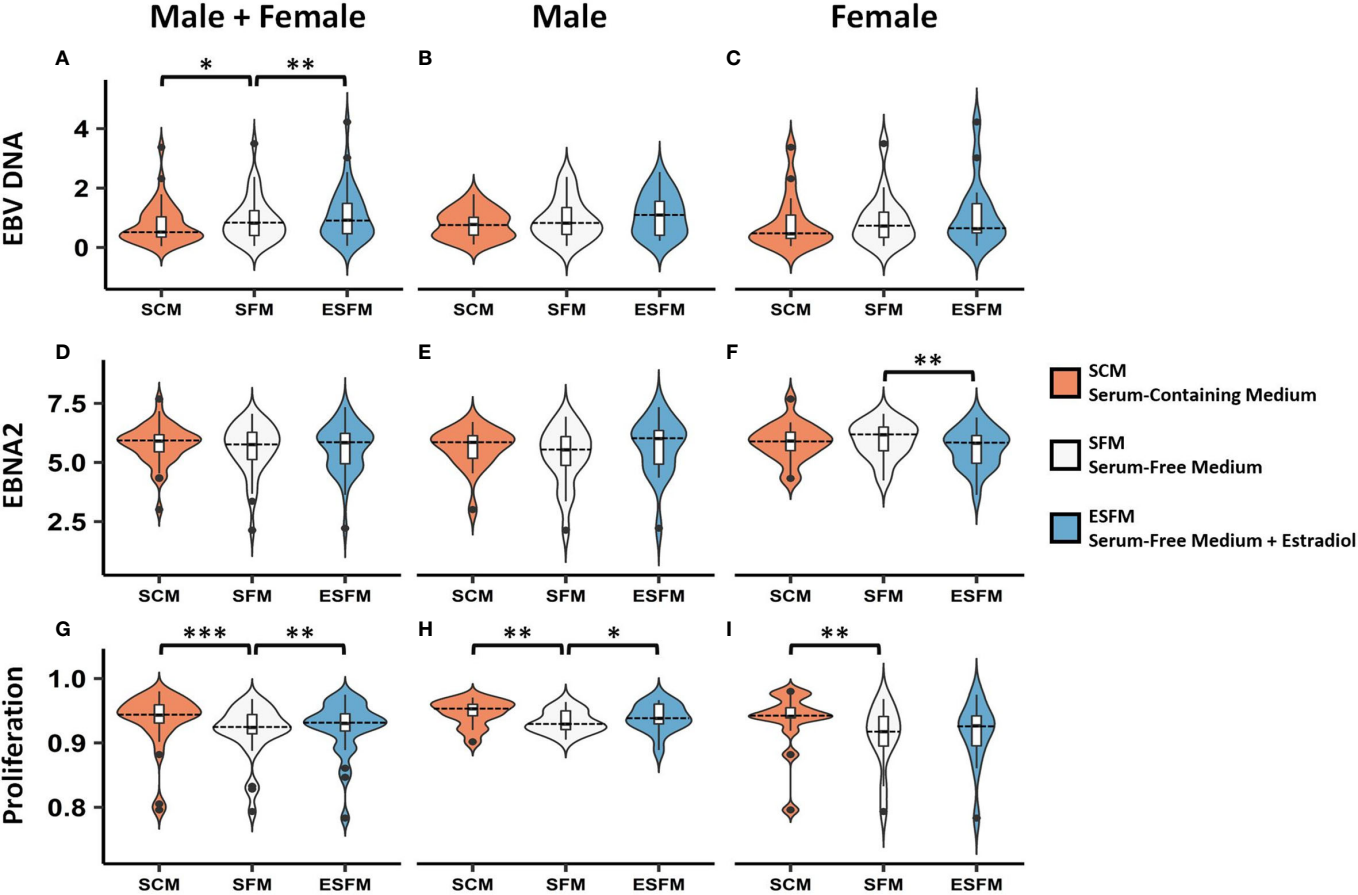
Effect of serum depletion and estradiol treatment of LCLs on EBV latency III. Top row, EBV DNA copy number. **(A)** Estradiol significantly increased EBV DNA copy number in male and female LCLs combined. Both male **(B)** and female **(C)** demonstrated insignificant increases in EBV DNA copy number in response to estradiol. Middle row, EBNA2 mRNA expression under different serum conditions. **(D)** Male and female LCLs combined, **(E)** Male LCLs, and **(F)** Female LCLs. EBNA2 expression was significantly reduced by estradiol in female LCLs. Bottom row, LCL proliferation. Removal of serum reduced proliferation in male and female LCLs combined **(G)**. Estradiol treatment increased cell proliferation significantly in male LCLs **(H)** but not significantly in female LCLs **(I)**. Male and female LCLs combined (n = 42), male LCLs (n = 21), and female LCLs (n = 21). EBNA2 expression detected by real-time PCR relative to RPL30 expression. Wilcoxon matched-pairs signed rank test performed (two-tailed). * < 0.05, ** < 0.01, *** < 0.001. SCM, serum-containing medium; SFM, serum-free medium; ESFM, serum-free medium with additional estradiol at a final concentration of 100 nM (see *Materials and Methods*).

To determine if estradiol or other components of the medium were affecting gender-biased gene expression, we investigated if serum depletion, or serum depleted medium augmented with estradiol, affected EBV latency III traits (EBNA2 expression, EBV DNA copy number, and cell proliferation) in a local LCL cohort (n = 42). LCLs were cultured in a typical serum-containing medium (SCM)—which contains numerous growth factors as well as the estrogenic pH indicator phenol red (50, 51)—or they were weaned from this into serum free medium (SFM) without phenol red, and then treated with estradiol 100 nM (ESFM), as outlined in **Figure 4**. **Figure 5** and **Supplementary Table 9** represent the effect of SCM, SFM and ESFM conditions on EBV traits in LCLs. EBV DNA copy number was significantly increased in SFM compared to SCM when male and female LCLs were combined for analysis (**Figure 5A**, p < 0.05); but this trait did not appear to be sex-biased, with both male and female LCLs responding similarly (**Figures 5B, C**). EBNA2 expression was significantly reduced by estradiol addition in female LCLs (ESFM compared to SFM, **Figure 5F**, p < 0.01). As expected, LCL proliferation was reduced when the medium was depleted of serum for both male and female LCLs (**Figures 5G–I**). Addition of estradiol to SFM increased proliferation in males, with a trend for increase seen in females (**Figures 5H, I**, p < 0.05, p < 0.1193). Taken together, these data indicate that LCL donor gender affects response to estradiol in EBV latency III.

### The Effect of Estradiol on MS Risk Gene Expression

**Figure 6:**
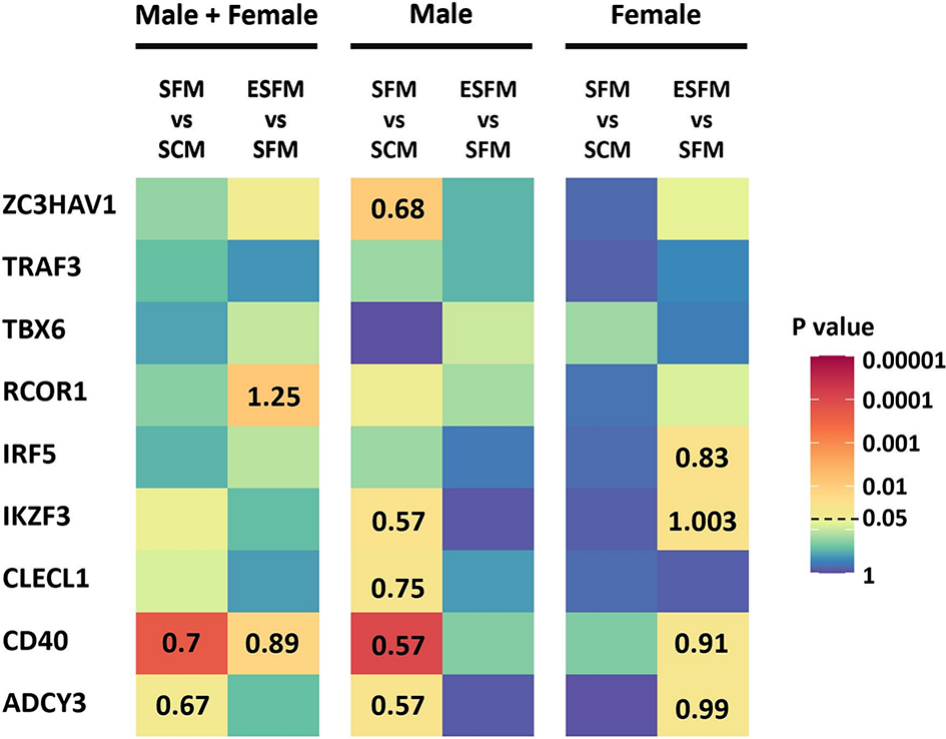
Effect of serum depletion and estradiol treatment of LCLs on the expression of nine MS risk genes. Left, LCLs are grouped to include both male and female LCLs combined (n = 42); center, male LCLs (n = 21); right, female LCLs (n = 21). Heatmap shows the statistical significance (p value) of the fold change in expression between serum-free medium and serum-containing medium (SFM *vs* SCM), or estradiol treated versus serum-free medium (ESFM *vs* SFM). For the comparisons that were statistically significant (p value less than 0.05), the fold changes are represented with numbers in the corresponding cells. Fold change values greater and less than one denotes increased and decreased expression levels, respectively. MS risk gene expression was measured by RT-qPCR. PCR was performed in duplicate with expression relative to RPL30. Expression relative to RPL30. Colors represent the p value as calculated by expression relative to RPL30. Wilcoxon matched-pairs signed rank test performed (two-tailed). Wilcoxon matched-pairs signed rank test performed (two-tailed). SCM, serum-containing medium; SFM, serum-free medium; ESFM, serum-free medium with additional estradiol at a final concentration of 100 nM (see *Materials and Methods*).

As we had identified several MS risk loci as being gender-biased eQTLs, with expression of some of their associated genes correlated with expression of ESR2, we next investigated whether expression of these genes was affected by serum depletion or estradiol supplementation of serum-free medium. Of the nine MS risk genes tested, five were significantly responsive to serum depletion in males, and none in females (CD40, ADCY3, ZC3HAV1, CLECL1 and IKZF3; **Figure 6**, p < 0.05). The response seen in males in serum-free medium was abolished by addition of estradiol. CD40, ADCY3 and IKZF3 were significantly altered by estradiol treatment in female but not male LCLs (**Figure 4**, p < 0.05). We did not detect any of the correlations of genotype with expression in any conditions seen in the much larger GEUVADIS cohort, presumably due to lack of statistical power (**Supplementary Table 10**).

## Discussion

In this study we investigated if there were gender differences in the interaction of MS risk genes with EBV latency III infection, and if these differences were affected by estradiol, to assess if the differences might underpin the increased susceptibility of females to MS. We identified several lines of evidence that point to a gender effect on the EBV latency III traits. Gene expression of the EBV latency III master regulator EBNA2 is highly negatively correlated with ESR2 expression in female LCLs, but not in males (**Figure 2**). The EBV latency gene LMP1, which is regulated by EBNA2, correlated negatively with ESR2 in male and female LCLs, but the statistical strength and effect size of this was greater in female LCLs. EBV DNA copy number was more positively correlated with ESR2 expression in female than male LCLs. There was also a gender effect on the association of MS risk loci with gene expression in LCLs. We identified 41 risk loci that were only eQTLs in one gender, 15 for males, and 26 for females, highlighting a marked difference between the MS risk SNP eQTL profiles of male and female LCLs. Of the 8 loci (8 MS risk SNPs associated with 9 MS risk genes) selected for further study, three were significantly correlated with ESR2 expression, in a sex-biased manner. Despite the observed sex biases, when comparing ESR2 expression only, no differences were detected between male and female LCLs (**Supplementary Figure 3**).

Standard culture medium for LCLs contains phenol red, a dye that is an estrogen analogue (50, 51), and FBS, which contains hormones and various cytokines. We depleted LCLs of serum and phenol red to eliminate estrogenic effects, which significantly reduced LCL proliferation and decreased EBV DNA copy number but did not alter EBNA2 mRNA in either gender. ESR2 expression was not altered (**Supplementary Figure 2**). Addition of estradiol did not affect EBV DNA copy number but reduced the difference in LCL proliferation caused by serum depletion. Expression of five (of nine tested) gender biased risk genes was reduced by serum depletion and restored by addition of estradiol. The response of these five genes to serum depletion was limited to males. However, estradiol addition did alter the expression of four of the gender bias risk genes in females. Together, these data indicate that estradiol regulates the expression of at least five of these MS risk genes in LCLs gender-specifically.

Epigenetic effects in B cells could potentially mediate the sexual dimorphism that we found in EBV latency III traits and the MS risk gene expression in this study. One study of CD19 B cells found that 3894 CpG sites were differentially methylated based on sex, which the authors postulated could be due to female sex hormones (63). EBV infection widely alters the epigenetic pattern (64, 65). EBV infected cells are reported to have a substantially altered methylome compared to their uninfected B cell counterparts. EBV infected B cell promoters tend to be hypomethylated (66, 67), particularly at sites corresponding to B cell biological pathways, when compared to resting B cells (67). Furthermore, we previously found that MS risk SNPs that are eQTLs in LCLs (LCLeQTLs) are more likely to be methylated than by chance (68). It is therefore plausible that in the current study that differential methylation in the genders could underpin the response to estrogen signaling observed in serum-containing medium. Response to estrogen in males and females is dose-dependent, and male immune cells have been reported to be more responsive *ex vivo* (69). We also found that expression of the MS risk genes was affected in males but not females by estrogen depletion. As well as being dependent on the concentration, response to estrogen may depend on existing methylation patterns and transcriptome differences that might not be significantly altered after 7 days of serum-depletion. Estrogen has also been reported to alter the genetic program of B cells to alter survival and activation, which can promote B cell autoreactivity (70), which is a mechanism which could alter EBV and MS risk gene expression patterns.

Estrogen could also interact with EBV. One research group have indicated that high estradiol levels (representative of the third trimester of pregnancy) reduced EBV reactivation (56, 57). Such a mechanism whereby estrogen controls EBV infection could explain the reduced MS disease activity typical of the third trimester of pregnancy, when estrogen is highest. Estrogen could affect B cell or EBV regulation and epigenetics directly through interaction at the transcriptomic level with genes such as the MS risk genes of this study. Four of the MS risk loci in this study are confirmed binding sites for EBNA2 with a total of five proximal genes associated with EBNA2 (TRAF3, RCOR1, CLECL1, CD40, and TBX6) (14). The IKZF3 locus is also targeted by EBNA2 but its transcription has not been reported as regulated by it to date. There was no association between the MS risk loci and ESR2 expression. Notably though, there are EBNA2 binding sites at potential regulatory sites within the ESR2 gene (13). This suggests regulation of gene expression through estrogen response may contribute to EBV dysregulation of the host transcriptome. Further work will determine whether estradiol alters EBNA2 binding at these sites to regulate these MS risk genes, or whether estrogen receptors directly colocalize with EBNA2 in a manner that could contribute to the sexual dimorphism found in this study.

The MS risk genes studied here function on pathways important in EBV immortalization (14), or in the case of ZC3HAV1 in host antiviral response, and therefore likely affect EBV survival in infected B cells in the host. We have previously proposed a model for how these genetic loci may alter MS susceptibility and/or progression through altering EBV pathogenesis in the host (14, 16). Demonstrating that EBNA2 is altered by estradiol treatment is therefore of importance. Estrogen could indirectly modulate expression of these MS risk genes in infected B cells *via* the regulation of EBNA2 dependent on gender. The treatment of EBV infection in MS has received some attention recently (11) and is therefore important to understand how estrogen or other hormones affect the EBV life cycle.

If estrogen drove increased genetic susceptibility to MS through affecting regulation of these gender biased risk loci, then we would expect the loci to be associated in one gender more than the other. This is not the case. Although both males and females are equally susceptible to EBV pathogenesis (41), they have different responses to the virus. Females, for example have higher titers of EBNA1 antibodies (42, 43). In the *in vitro* context female LCLs grow about 7% slower than male LCLs (71), which could be due to the estrogenic culture conditions. Therefore, we suggest that estrogen may drive gender differences in susceptibility by altering the host response to EBV. The gender specific genetic differences we observed in risk loci eQTLs may indicate these genes are important in the host response, but affect progression rather than susceptibility. This hypothesis is supported by evidence of a gender effect in the correlation between the age of IM and the development of MS (44). Females who had IM earlier in life developed MS slower than males who had IM earlier in life, and slower than females who had IM beyond puberty (44). As such, the effect of EBV infection on MS may be tractable to therapeutic intervention.

To detect eQTL differences in the MS risk loci in response to estradiol, larger cohort sizes are needed. Response could also be affected by other time points and concentrations of estradiol, stages of the EBV life cycle examined, switching between stages, other hormones, the relevance of *in vitro* models, and by types of estrogen. Additionally, next-generation sequencing, such as with RNA-seq and ChIP-seq, and immunophenotyping would provide a more comprehensive assessment of sex hormone response in EBV infection. This study detected gender associations in LCLs, a well-established model of the EBV latency III stage of infection, and tested the effects of estrogen in LCLs. It is possible that these observed gender differences and responses may be different in EBV infected B cells *in vivo*. Therefore, testing of sex hormones in an EBV animal model, such as described by Wirtz and colleagues (72), will more clearly define the role of hormones in both MS and EBV infection.

We conclude that estradiol treatment alters EBV latency III functions and regulates MS risk genes differently between males and females. The consequences of this on EBV infection and MS pathogenesis need to be determined.

## Data Availability Statement

The original contributions presented in the study are included in the article/**Supplementary Material**. Further inquiries can be directed to the corresponding author.

## Ethics Statement

The studies involving human participants were reviewed and approved by Westmead Hospital Human Research Ethics Committee. The participants provided their written informed consent to participate in this study.

## Author Contributions

DB, AA, JK, and GP conceived the project. JK conducted all laboratory experiments and analysis, with the exception of the preliminary data in **Supplementary Figure 1**, which was conducted by NF. AA analyzed the publicly available datasets that were used here. AA and JK generated figures and tables. SSc provided methodology advice and optimized the preparation of the serum-free media used for this study. GP assisted in data analysis. JK, AA, and DB prepared the manuscript. All authors contributed to the article and approved the submitted version.

## Funding

JK and AA were funded by an Australian Government Research Training Program (RTP) scholarship. JK was also supported by a University of Sydney Postgraduate Merit Award and Westmead Institute for Medical Research top-up supported by the Leece family. AA was also supported by a Du Pre grant from the Multiple Sclerosis International Federation, and the EBV Molecular biology Lab, Westmead Institute for Medical Research, University of Sydney. The study was supported by Project and Incubator grants from MS Research Australia (numbers 19-0640 and 19-0627, respectively). GP was supported by an MS Research Australia Postdoctoral Fellowship and a Trish Multiple Sclerosis Research Foundation Project Grant. DB was supported by an NHMRC Senior Research Fellowship. Funding bodies had no role in the design of the study, analysis, or interpretation of data or in writing the manuscript.

## Conflict of Interest

The authors declare that the research was conducted in the absence of any commercial or financial relationships that could be construed as a potential conflict of interest.

## Publisher’s Note

All claims expressed in this article are solely those of the authors and do not necessarily represent those of their affiliated organizations, or those of the publisher, the editors and the reviewers. Any product that may be evaluated in this article, or claim that may be made by its manufacturer, is not guaranteed or endorsed by the publisher.
