# Supplementary material for: Gender and the Sex Hormone Estradiol Affect Multiple Sclerosis Risk Gene Expression in Epstein-Barr Virus-Infected B Cells
Source: Front Immunol. 2021 Sep 8;12:732694. doi: 10.3389/fimmu.2021.732694 (PMC8455923; doi:10.3389/fimmu.2021.732694)
Supplement: Supplementary file 1 [file DataSheet_1.docx]

Supplementary Material


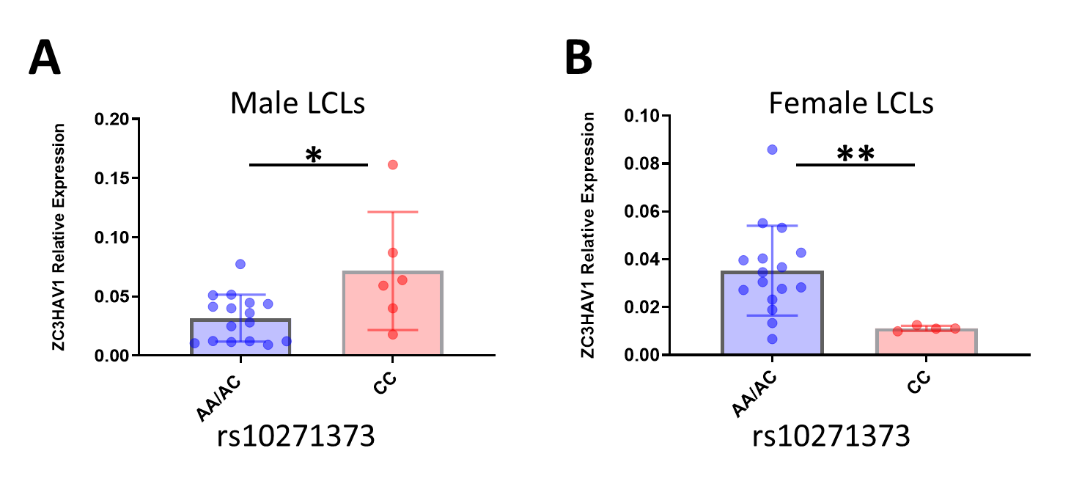


**Supplementary Figure 1.** The effect of genotype on the expression of ZC3HAV1 in LCLs in male and female LCLs in a preliminary analysis from the WIMR cohort. (A) The MS risk allele C increases ZC3HAV1 expression in male LCLs significantly (n = 22), but in female LCLs it has the opposite effect, significantly reducing expression of ZC3HAV1 (n = 20), indicating a gender-dependent host EBV response. * < 0.05, **<0.01. Gene expression relative to RPL30 expression. Mann-Whitney test performed for unpaired samples. LCLs were cultured in serum-containing medium as described in Materials and Methods.

**
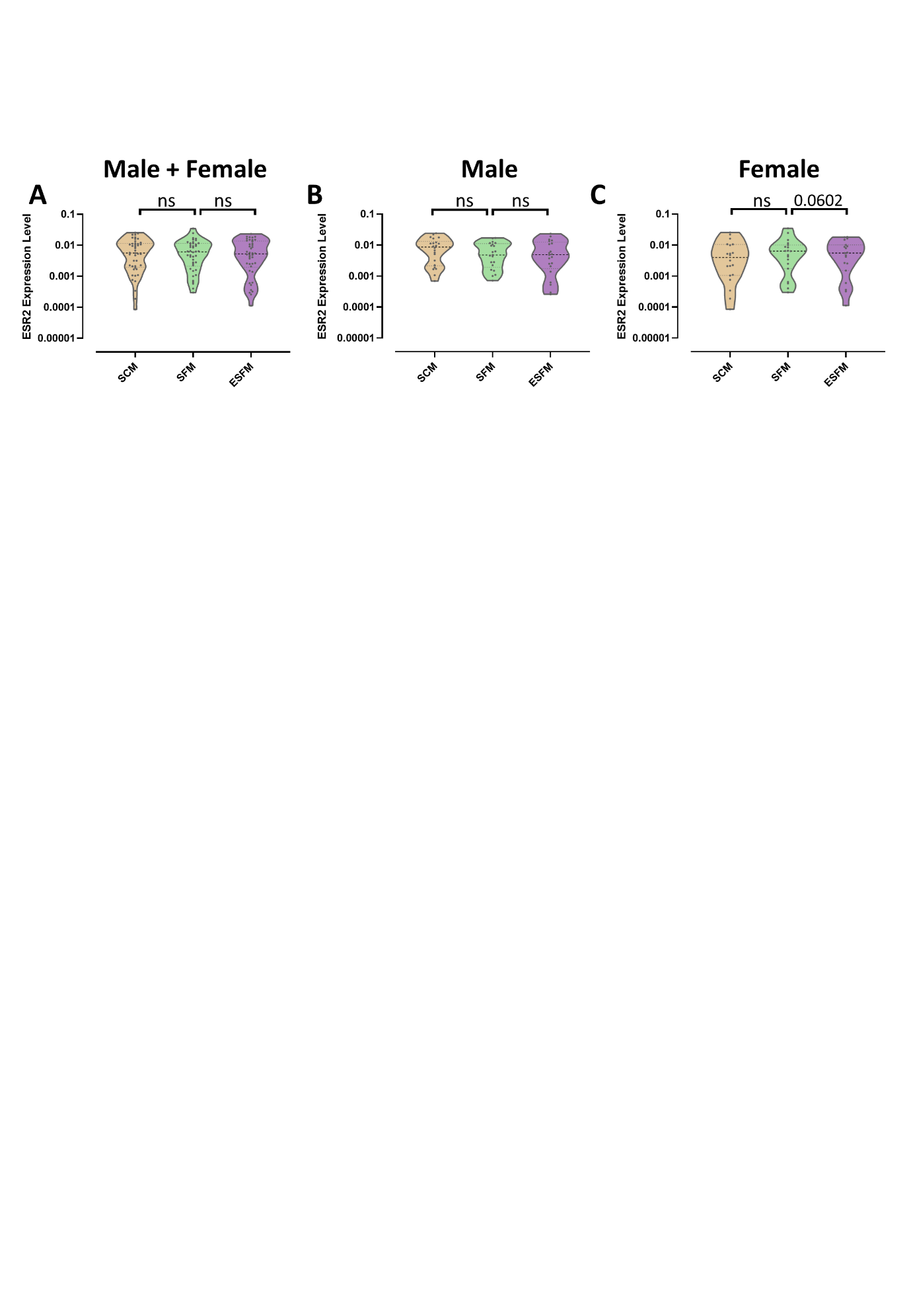
**

**Supplementary Figure 2.** Effect of serum depletion and estradiol treatment of LCLs on Estrogen Receptor 2 (ESR2) gene expression. ESR2 mRNA expression under different serum conditions for (A) Male and female LCLs combined (n = 42), (B) Male LCLs (n = 21), and (C) Female LCLs (n = 21). ESR2 expression was not significantly affected by serum depletion or estradiol treatment. ESR2 Expression detected by real-time PCR relative to RPL30 expression. Wilcoxon matched-pairs signed rank test performed (two-tailed). ns denotes statistically non-significant with a p value of greater than 0.05. SCM, serum-containing medium; SFM, serum-free medium; ESFM, serum-free medium with additional estradiol at a final concentration of 100 nM (see Materials and Methods).

**
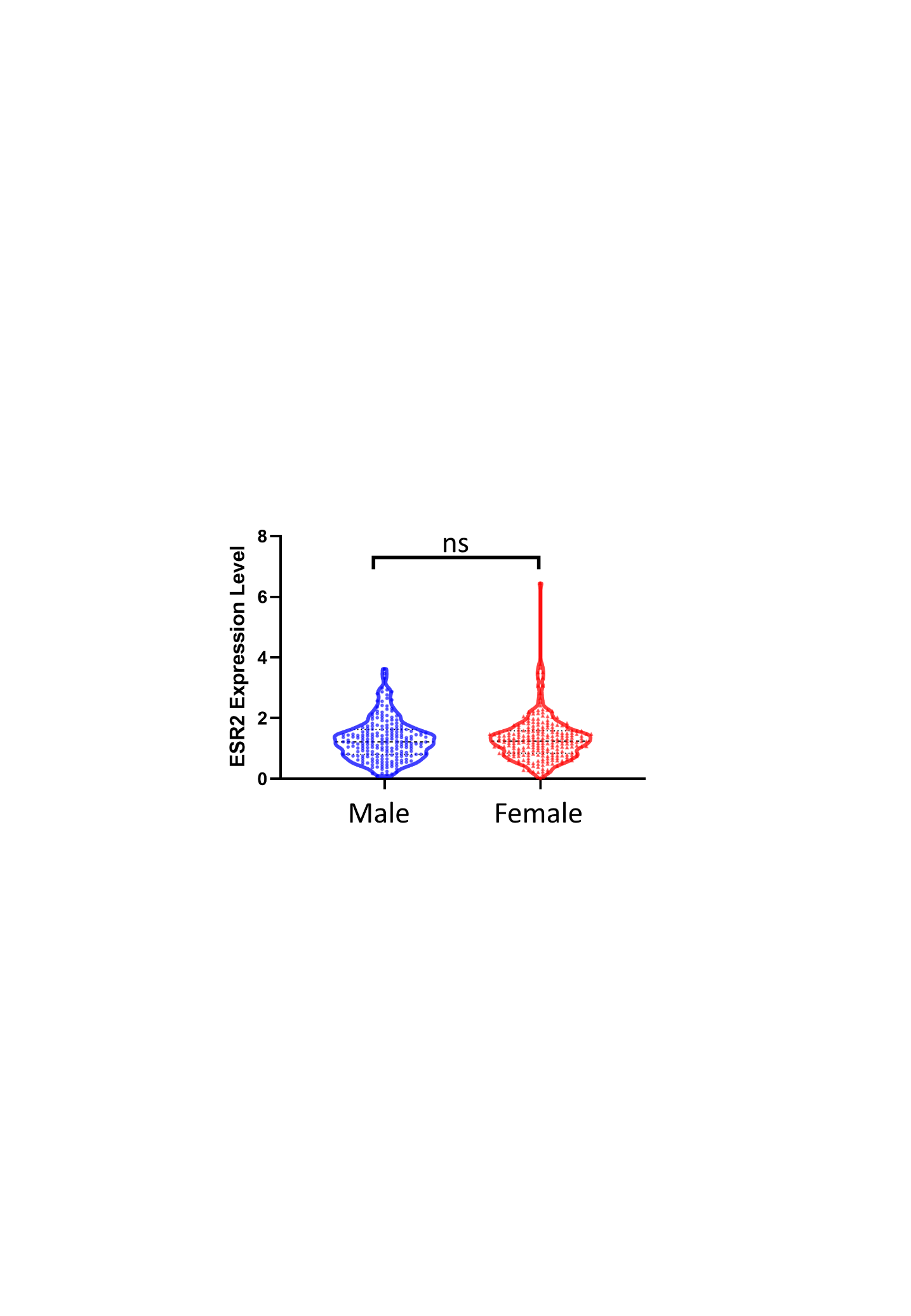
**

**Supplementary Figure 3.** Comparison of ESR2 expression in male and female LCLs from the the GEUVADIS dataset. A Mann-Whitney test was performed to compare the groups. ns denotes statistically non-significant with a p value of greater than 0.05. The GEUVADIS LCL cohort consisted of 358 European samples using RNA-seq including 187 female and 171 male LCLs.

**Supplementary Tables**

**Supplementary Table 1:** Supplementary Table 1: 196 MS risk SNPs and 216 genes in proximity of them totaling 229 MS risk SNP:gene pairs tested for eQTL association.

**Supplementary Table 2:** Serum-free Medium (SFM) Preparation.

**Supplementary Table 3:** Gene Expression Probes.

**Supplementary Table 4:** Primer pairs.

**Supplementary Table 5:** Genotyping Probes.

**Supplementary Table 6:** The 73 MS risk SNP:gene pair eQTLs in the female and male LCLs separately and both together using 373 European LCLs from the GEUVADIS dataset.

**Supplementary Table 7:** The correlation between Estrogen receptor 2 (ESR2), Estrogen receptor 1 (ESR1) and Androgen receptor (AR) expression level and phenotypic markers of EBV latency III (EBNA2 and EBV DNA copy number level). Female + Male, LCLs are grouped to include both male and female LCLs; Male, male LCLs; Female, female LCLs. The correlations were calculated using Spearman's rank correlation coefficient. Note: progesterone receptor (NR3C3) was undetected.

**Supplementary Table 8:** The correlation of selected MS risk genes with Estrogen receptor 2 (ESR2) and the EBV latency III traits EBNA2 and EBV DNA copy number (DNA) of donor matched LCLs from the GEUVADIS dataset. Female + Male, LCLs are grouped to include both male and female LCLs; Male, male LCLs; Female, female LCLs. The correlations were calculated using Spearman's rank correlation coefficient.

**Supplementary Table 9:** Effect of serum depletion and estradiol treatment of LCLs on the expression of nine MS risk genes. Female + Male, LCLs are grouped to include both male and female LCLs; Male, male LCLs; Female, female LCLs. Fold change was calculated based on the changes in the expression level in serum-free medium compared to serum-containing medium, or estradiol treated versus serum-free medium. Fold change equal to one means no change. Wilcoxon matched-pairs signed rank test performed (two-tailed). Wilcoxon matched-pairs signed rank test performed (two-tailed).

**Supplementary Table 10:** The eQTL effect of seven MS risk loci on eight genes in LCLs of the WIMR cohort in serum-containing medium (SCM). Data shown is the analysis for Male and Female LCLs combined (n = 42), Male LCLs (n = 21), and Female LCLs (n = 21). Note: the ADCY3 locus rs11125803 was excluded due to a lack of genotype data.
